# Feasibility and Safety of Superomedial Pedicle for Breast Reduction in Geriatric Patients

**DOI:** 10.1007/s00266-024-03859-9

**Published:** 2024-03-15

**Authors:** Alp Ercan

**Affiliations:** Cerrahpasa Medical Faculty, Istanbul University-Cerrahpasa, Yesilkoy Cad./Bakirkoy, Istanbul, Turkey

**Keywords:** Breast reduction surgery, Reduction mammoplasty, Superomedial pedicle, Elderly women, İncreasing age, İnferior pedicle

## Abstract

**Introduction:**

Historically, inferior breast reduction is more commonly performed overall and this applies to the elder population. No study to this date has compared whether there is any difference in complications and overall safety between when using superomedial pedicle and inferior pedicle in geriatric patients and furthermore whether the safety profile of superomedial pedicle differs when compared to general population.

**Methods:**

Patient files of women who had undergone breast reduction by a single surgeon over a 9 year period (2015–2023) was reviewed retrospectively. Patients over 65 years old at the time of surgery were selected as the main study group. Results were compared to a control group aged 65 years and younger consisting of 136 patients, who also had a breast reduction by the same surgeon.

**Results:**

Fifty-four women met the inclusion criteria for the study group and they were further broken down into two subgroups; inferior and superomedial pedicle groups with 25 and 29 patients into each group, respectively. The mean age at the time of the operation was 67.8 years. Geriatric group had more significant comorbidities (37% vs. 9%, *p*<0.05). Looking solely on patients undergone superomedial pedicle breast reduction, OR times were similar between two age groups and hospital stay was slightly longer in the geriatric population albeit statistically insignificant. The average weight of specimens resected from each breast was 592.4 gr in geriatric population and slightly higher in the younger population with an average weight of 624 grams (*p*=0.27). Two women in the geriatric group and  six women in the  non-geriatric group developed major complications where superomedial pedicle was utilized, no meaningful difference was seen when major complications were compared (*p*=0.24). On the other hand, minor complications were significantly higher in the geriatric population compared to the younger cohort regarding superomedial pedicle reductions (*p*=0.02). ‘Satisfaction with breasts’ scores of BreastQ from the superomedial breast reduction subgroup was slightly higher than inferior pedicle breast reduction subgroup in geriatric population and it was statistically significant (0.032).

**Conclusion:**

Safety margins and satisfaction scores of superomedial pedicled breast reduction in geriatric patients seem similar to their younger counterparts. Furthermore, with similar complication rates and with its slightly higher ‘Satisfaction with breasts’  scores  when compared to inferior pedicle, superomedial pedicled breast reduction technique can be utilized without reservation in geriatric candidates for breast reduction.

**Level of Evidence IV:**

This journal requires that authors assign a level of evidence to each article. For a full description of these Evidence-Based Medicine ratings, please refer to the Table of Contents or the online Instructions to Authors www.springer.com/00266.

## Introductıon

Breast reduction, being a highly requested esthetic operation for women of various ages, is also a functionally relieving operation for many of the patients who had to carry a significant and debilitating weight for many years. Timing and reasoning behind seeking for a breast reduction changes from person to person and these can range from purely esthetic reasons to purely physiotherapeutic reasons [1, 2]. Breasts typically don't get larger in volume with age but they tend to sag significantly especially in child-bearing women. Naturally with age our physical attributes diminish and therefore it is not unusual for older women to ask for breast reduction even for supposedly smaller breasts complaining of their weight. As the socially active age has got higher in contemporary times and and also people in general continue to be a participant of the workforce longer than the past, more women in their late 60s and older become interested in breast reduction. Although breast reduction is considered a safe and reliable surgery in older women, there are few studies investigating specific circumstances in geriatric population such as the utility of different pedicles or length of OR time or if satisfaction rates after reduction surgery are similar to the general population [3, 4]. Despite the fact that pain relief and postural improvement are generally considered primary goals for older women who seek out breast reduction and treatment plans are drawn out accordingly, this can be a misconception as classically accepted age standards are shifting [5].

As body image becoming increasingly important in the elderly much like facial rejuvenation, more contemporary breast reduction techniques such as superior pedicle or superomedial pedicle can be utilized rather than more classical techniques like inferior pedicled breast reduction, as contemporary techniques may have answers to several esthetic concerns of classical techniques. Although superomedial pedicle is proven to be equally safe with similar complication rates when compared with inferior pedicle, its reliability and safety is still uninvestigated in geriatric population. To this date, only few studies specifically address age related issues concerning symptoms, complication rates and patient satisfaction of breast reduction in the geriatric population. In this study, we aim to compare superomedial pedicle to inferior pedicle regarding complications and patient satisfaction specificly in the geriatric population. As a secondary objective we wanted to find out if safety margin and satisfaction rates of superomedial pedicled breast reduction are similar to the younger population.

## Methods

All women aged 65 years and older who had breast reduction surgery by the primary surgeon between 2015 and 2023 were reviewed retrospectively. Patients with unilateral breast reductions, e.g., matching procedures after contralateral breast reconstruction, patients who had an incidental malignancy in the breast specimen and patients with less than 6 months follow-up periods were excluded. All the patients included in the study underwent screening for breast cancer using an imaging technique appopriate for their age prior to surgery. Patients were selected through the electronic database. Another group of 136 women aged 65 and younger was selected for comparison, who also received breast reduction surgery throughout the same time period and with similar inclusion/exclusion criteria. The operative procedures included supero-medial pedicles and inferior pedicles both with inverted-T scars. To construct similar groups, vertical scar patients and patients who had a superior pedicle breast reduction were omitted from the younger cohort as well. Ethical approval was provided by the Institutional ethics committee. Complications recorded were classified and grouped in significance (minor and major complications). Superficial wound infections e.g., cellulites not necessitating IV antibiotherapy, palpable fat necrosis and small seromas and hematomas that do not need surgery and minor wound dehiscence without a need for procedural intervention were all classified as minor complications. Major complications involve deep tissue infection necessitating IV antibiotherapy or internalization, hematomas with immediate return to OR, deep venous thrombosis, a large wound dehiscence needing a secondary closure and any extent of NAC loss. Minor superficial wounds that don't need any attention, local suture exposures and small fat necrosis deposits that are hard to palpate were not classified as valid complications. In addition, patient satisfaction was measured by a validated questionnaire and results were compared to the data set of younger patients. Validated questionnaire was done after a minimum of 6 months of follow-up period. BreastQ was was used as it has been shown to provide good test/re-test reliability and validity and has been successfully used in multiple studies.

Statistical analysis was performed with SPSS ver 25. Comparison of normally distributed, continuous data between two groups was performed with unpaired t-test, categorical data with fisher’s exact test.

## Results

Complication rates and patient satisfaction of geriatric women undergoing breast reduction using inferior and superomedial pedicle were analyzed and compared to a cohort of younger patients who had a breast reduction with similar pedicles by the same surgeon in the same time period (Tables 1, 2, 3 and 4). Mean follow-up time after surgery at the time of the BREASTQ questionnaire was 2,7 years. As standart protocol, all the resected breast specimens were sent for pathological work-up. No incidental malignancy was detected in the breast resection specimens of the study group during the routine pathological work-up. Geriatric population consisted of 54 patients with a mean age of 67.8 years (youngest 66–oldest 76) and younger cohort had a mean age of 41,2 years (youngest 21–oldest 60). BMI was slightly higher in the younger cohort without any statistical significance (young: 28.6 kg/m^2^; elderly: 28.2 kg/m^2^, *p*=0.67). The average overall resection weight per breast in the geriatric cohort was 592 g (±341 g) with slightly higher resection weights in younger women (624 g ± 380 g ). Operative time was not significantly different neither between the different age groups (148 in geriatric vs 153 minutes in young, *p* = 0.374) nor between two subgroups of distinct pedicles (142 in superomedial vs 151 inferior, *p* = 0.12). Despite not being statistically meaningful, OR time for superomedial pedicle patients was slightly shorter than inferior pedicle patients. Geriatric women naturally had significantly more comorbidities (35 vs. 9; *p*<0.05), albeit smoking was more common in the young (3 vs 32; *p*=0.032). Comorbidities such as HT (Hypertension), CAD (Coronary artery disease) and DM (Diabetes mellitus) were higher in the geriatric group with CAD being the most distinct entity. CAD (in the form of myocardial infarction and angina) was observed in just 8 patients in nongeriatric (5,8%), whereas geriatric patients had a total of 19 (35%) (5,8% vs 35%, *p* < 0.05).

**Table 1:** Demographics, peroperative and operative data of the patients

	Geriatric (*n*=54)		Non-geriatric (*n*=136)	
	Inferior pedicle	Superomedial pedicle	Inferior pedicle	Superomedial pedicle
Number of patients	25	29	44	92
Age	67.4	68.1	43.2	39.6
BMI (kg/m^2^)	28.1	28.4	29	28.3
Diabets Mellitus	7	7	1	0
Hypertension	8	12	9	8
Smoking	2	1	18	14
Coronary Artery Disease	7	12	5	3
Peripheral Artery Disease	1	0	1	1
Amount of resected breast tissue (grams)	602	583	659	594
Operative length (min)	151	142	172	144
Length of stay (days)	1.17	1.12	1.08	0.92
Major complications	2	2	5	6
Minor complications	6	9	11	17

Geriatric subgroup A consisting of superomedial pedicle reductions and subgroup B consisting of inferior pedicle had no statistical difference in preoperative (BMI,comorbidity,age, etc.) or operative (OR time, LOS, resected breast tissue weight etc.) factors. A detailed list, specifying each data can be found in the accompanying table (Tables 1 and 3).

There were no statistically significant differences in major or minor complications when superomedial pedicle and inferior pedicle subgroups were compared in the geriatric cohort. Looking solely on patients who had a superomedial pedicle breast reduction, in the geriatric population we encountered minor complications in 9 patients out of a group of 29 (32%), and in the younger cohort we observed 17 minor complications out of a group of 92 (18,5%)

Most common minor complications were small incision dehiscence followed by cellulitis managed by oral antibiotherapy and palpable fat necrosis. There were two patients with major complications in superomedial pedicle breast reduction subgroup of geriatric patients (6,8%), one hematoma with emergency take-back and one vertical scar dehiscence that needed revision compared to 6 patients with major complications in the younger cohort (6,5%). Two patients had major complications in the inferior pedicle breast reduction group in geriatric patients which was a deep infection that called for IV antibiotherapy and the other was again a vertical incision dehiscence that needed revision. Apart from two major dehiscence patients stated above, none of the patients in the geriatric population asked for a revision of the surgery. Another interesting outcome from the study was that even though BMI had no impact on major complications in the geriatric group, its impact on minor complications was statistically significant (*p* 0,003).

**Fig. 1:**
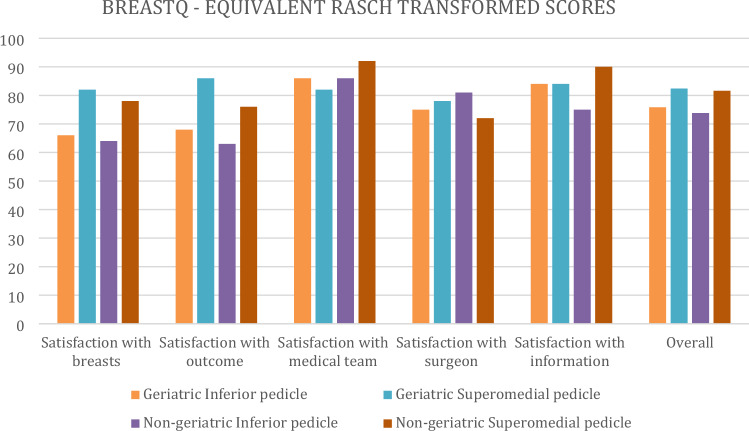
BreastQ subsection score comparison between different subgroups

BreastQ scores were slightly higher in the overall geriatric cohort when compared with younger population without any statistical significance. A detailed comparison was made between inferior pedicle and superomedial pedicle breast reduction focusing on geriatric patients. Superomedial subgroup scored higher than inferior pedicle subgroup on 'SATISFACTION WITH BREASTS' subsection of BREAST-Q- REDUCTION MODULE VERSION 2.0, and the difference was statistically significant (*p*=0,032) (Tables 2, 3, 4 and Fig. 1).

**Table 2:** BreastQ subsection scores of subgroups

BreastQ Subsection	Geriatric (*n*=54)						Non-geriatric (*n*=136)					
	Inferior pedicle			Superomedial pedicle			Inferior pedicle			Superomedial pedicle		
	Mean Score	Sum Score	ERTS (0–100)	Mean Score	Sum Score	ERTS (0–100)	Mean Score	Sum Score	ERTS (0–100)	Mean Score	Sum Score	ERTS (0–100)
Satisfaction with breasts	3.306	43	66	3.71	49	82	3.28	42	64	3.68	48	78
Satisfaction with outcome	2.65	21	68	2.87	23	86	2.55	20	63	2.76	22	76
Satisfaction with nipple	3.2			3.4			2.8			3.2		
Satisfaction with medical team	3.67	26	86	3.57	25	82	3.7	26	86	3.86	27	92
Satisfaction with surgeon	3.58	43	75	3.65	44	78	3.74	45	81	3.49	42	72
Satisfaction with information	3.8	50	84	3.81	50	84	3.69	48	75	3.96	51	90
Overall			75,8			82.4			73.8			81.6

**Table 3:** Complication and BreastQ score comparison of superomedial pedicle and inferior pedicle in geriatric patients

Parameters		Geriatric group		*p*
		Superomedial pedicle	Inferior pedicle	
BREASQ Overall Mean		82.40	75.8	0.21
BREASTQ scores—Satisfaction with breasts	Mean Score	3.71	3.31	0.032**
	Sum Score	49.00	43.00	
	ERTS (1–100)	82.00	66.00	
Major complications (%)		6.80	8.00	0.46
Minor complications (%)		31.00	24.00	0.06
Operative length (min)		142.00	151.00	0.13
Length of stay (days)		1.12	1.17	0.28

**signifies statistically significant values

**Table 4:** Complication and BreastQ score comparison of geriatric and non-geriatric patients who had superomedial pedicle breast reduction

Parameters		Superomedial Pedicle		*p*
		Geriatric	Non-geriatric	
BREASTQ Overall Mean of Subsections		82.4	81.6	0.57
BREASTQ scores - Satisfaction with breasts	Mean Score Per Question	3.71	3.68	0.42
	Sum Total Score	49	48	
	ERTS (1–100)	82	78	
Major complications (%)		6,8	6,5	0.24
Minor complications (%)		31	18	0.02***
Operative length (min)		142	144	0.6
Length of stay (days)		1.12	0.92	0.28

***signifies statistically significant values

**Fig. 2:**
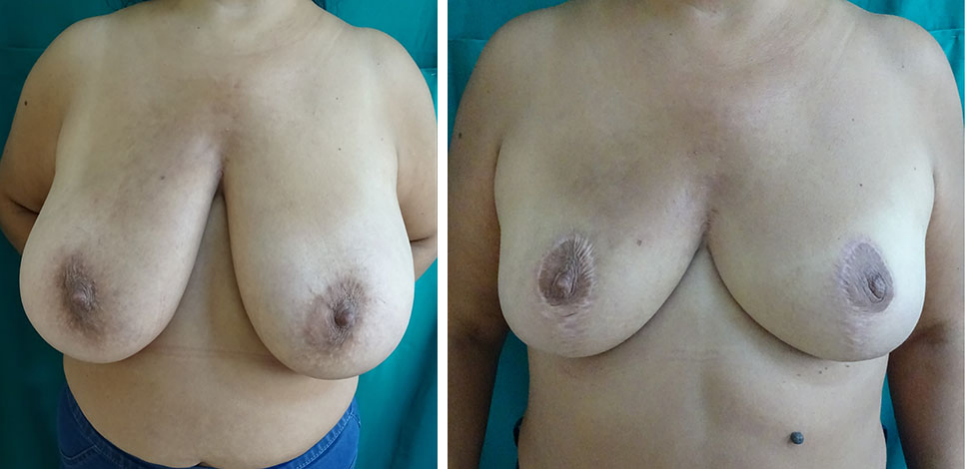
Preoperative and postoperative photos (13 months after surgery) of 67 years old patient with a strernal notch-to-nipple distance of 41&42 cms (L&R) and accompanying diabetes. She had a superomedial pedicled breast reduction and had minor incision dehischence at the upper segment of the vertical scar on the right breast which was managed in the outpatient setting. Despite the complication and not ideal scarring, she gave the highest score for satisfaction with breasts section

**Fig. 3:**
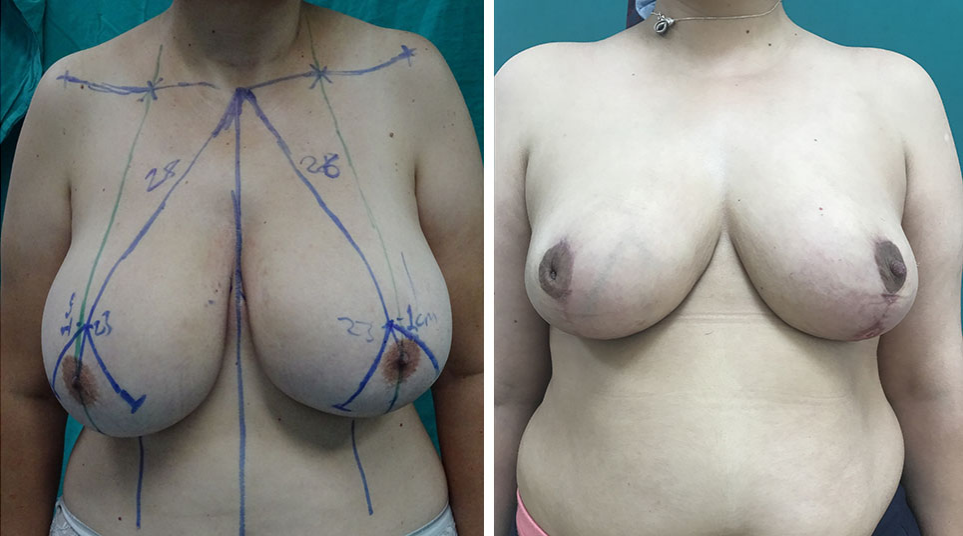
Preoperative and postoperative photos (9 months after surgery) of 45 years old patient with a strernal notch-to-nipple distance of 28 cms bilaterally. She had a superomedial pedicled breast reduction and had minor incision dehischence at the middle segment of the vertical scar on the left breast which was managed in the outpatient setting. Despite the complication and not ideal scarring, she gave the highest score for satisfaction from breast section.

One geriatric and one non-geriatric patient’s, who had superomedial pedicled breast reduction, preoperative and postoperative pictures can be seen in Figs. 2 and 3. Both patients gave the highest scores possible on the 'SATISFACTION WITH BREASTS' subsection despite problematic healing during follow-up and the obvious problems about scarring.

## Dıscussıon

As we are facing an aging population that is living longer, staying both socially and economically active for longer; the age range of patients looking up to have cosmetic surgery is widening as well [5–8]. This directly correlates to higher numbers of women in geriatric population asking for breast reduction. It is initially believed by the previous generation of plastic surgeons that are predominantly male that elder women over a certain age prioritized orthopedic and physiological gains ahead of esthetic improvement when it comes to breast reduction [9]. Even the primary author had the same misconception of this sexual and age-based bias. But for many of the socially active women of elder age, esthetic improvement is at least as important as orthopedic improvements and therefore an operation plan should be drawn accordingly [1, 10]

Inferior pedicle is still the most commonly used breast reduction technique according to U.S. numbers [11]. It is a robust pedicle and a reliable time-proven technique for breast reduction which also allows to offload as much weight as the surgeon wants. It is thought that resecting higher amounts of breast tissue would be beneficial to this particular patient group as it would directly decrease the weight that the patient will carry and therefore would potentially decrease the neck/shoulder/back pain. It is also considered a 'safer' option for breast reduction anecdotally and many surgeons prefer inferior pedicle over the other options when a patient has a significant comorbidity such as diabetes, smoking history or in our case if the said patient is above a certain age limit. This approach does not stem from any validated data but rather personal preference and experience [12]. Even though an inferior pedicle is considered the standard approach for breast reduction for a considerable majority of plastic surgeons, there are certain esthetic concerns such as the boxy appearance of breasts, empty cleavage and being more prone to bottoming out [13]. On the other hand, a superomedial pedicle is a more contemporary technique which can provide a subjectivelly better breast shape and a more prominent cleavage due to the nature of its pedicle design and pedicle movement. For these particular reasons, it became a popular option albeit not used as commonly as inferior pedicle. Initially being considered not as safe and reliable as inferior pedicle, it has been shown to be as reliable as inferior pedicle as many recent studies contradicting unwarranted safety issues have been published in the last two decades [14, 15]. Despite its proven general safety, we believe superomedial pedicle is still underused in geriatric patients. As most studies have been performed in relatively younger women, little is known about complications and the safety profile of superomedial pedicle in elderly women.

When complications are compared within geriatric patients, both minor and major complications didn't differ between superomedial pedicle and inferior pedicle (Tables 3 and 5). Regarding specifically the superomedial pedicle, there was a noticeable increase in minor complications in geriatric patients compared to the younger cohort (32% vs 18,5% and *p* 0,03). We believe this increase in minor complications can be due to the overall decrease in wound healing capacity in the elder population and the slight increase in accompanying comorbidities compared to younger population that can potentially impair wound healing. The important finding was, there was no statistically significant difference in major complications between geriatric patients and younger patients when a superomedial pedicle was used. In our opinion, this data can attest to the safety of superomedial pedicle in geriatric population.

**Table 5:** List of all complications in the study cohorts

Complications	Groups			
	Inferior pedicle (geriatric subgroup) *n*=25	Superomedial pedicle (geriatric subgroup) *n*=29	Inferior pedicle (non-geriatric subgroup) *n*=44	Superomedial pedicle (non-geriatric subgroup) *n*=92
Hematoma with immediate take-back	0 (0%)	1 (%3, 4)	1 (2, 2%)	2 (2, 1%)
Major wound dehischence necessitating revision	1 (4%)	1 (%3, 4)	1 (2, 2%)	1 (1%)
Deep tissue infection managed by IV antibiotherapy	1 (4%)	0	3 (6, 8%)	2 (2, 1%)
Nipple areola complex loss (partial/complete)	0	0	0	1 (1%)
Deep venous thrombosis	0	0	0	0
Superfical tissue infection managed by oral antibiotherapy in out-patient setting	2 (8%)	2 (6, 7%)	5 (11, 3%)	4 (4, 3%)
Minor wound dehischence managed without intervention	3 (12%)	5 (17%)	3 (6, 8%)	9 (9, 7%)
Palpable fat necrosis	1 (4%)	2 (6,7%)	3 (6, 8%)	4 (4, 3%)

Breast reduction is a very satisfactory and life altering surgery for many women and there are multiple studies showing high BREASTQ scores after breast reduction surgeries. Liao et al showed that elder women also scored high BREASTQ scores after breast reduction surgeries and even though this study didn't specifically looked at the geriatric population, their cut-off at 60 years of age can make a solid argument [16–18]. Overall high satisfaction rate of the geriatric group in our study supports this argument. Even though we haven't seen a statistically significant difference in overall BREASTQ scores between superomedial pedicle group and inferior pedicle group in geriatric patients, we saw superomedial pedicle group score higher numbers in 'SATISFACTION WITH BREASTS' subsection of BREAST-Q- REDUCTION MODULE VERSION 2.0 compared to inferior pedicle group and difference was statistically significant (*p*=0,038). Looking into the specific questions that contribute to this difference, we have seen scores for questions; 'The shape of your breasts when you are not wearing a bra?' and 'How normal your breasts look?' were meaningfully higher in the superomedial pedicle group (3,45 and 3,78, respectively) compared to inferior pedicle group (3,02 and 3,34, respectively). In our experience, superomedial pedicle can give a more pleasing and natural shape in the long term and the difference in these scores can be related to that. Another peculiar outcome from the study is that even though minor complications are higher in the geriatric cohort compared to the younger patients, BREASTQ scores of geriatric cohort are still as high as the younger cohort. Although their effect on quality of life is not as dramatic as major complications, minor complications can still be a hindrance for the patients and may affect their satisfaction from the surgery. The fact that this higher percentage of minor complications is not affecting the satisfaction scores of elder patients may be due to their higher tolerance to problems that usually comes with accumulated life experience after a certain age.

Limitations of this study is its retrospective nature and relatively low number of patients included in the study. As patients' numbers are on the lower side, the number of major complications encountered are quite few. In both the geriatric (*n*=54) and the control group (*n*=136) , we haven't seen any DVT and therefore we can't make a point for or against another major study by Gustavo et al about complication profiles of breast reduction patients who subsequently developed a DVT [19].

## Conclusıon

A plastic surgeon should always bear in mind that breast reduction surgery in geriatric population must address specific age-related expectations and complications, but it shouldn't mean that the only primary objective of the surgery is to relieve the patient of their postural and orthopedic problems. As we acknowledge esthetic concerns of elderly women for an optimal result, and treat them with similar high esthetic standards as we always do with younger women, a consistent high satisfaction rate from the surgery will follow. With similar complication rates, with slightly lower operative times and higher satisfaction rates than the classical inferior pedicle technique, superomedial pedicled breast reduction appears as a safe and reliable procedure in geriatric population. Despite our statistically significant data, it should be stressed that our study population is comparatively smaller to similar studies and there is a more prominent variety of potential comorbidities that may dictate other options for the elder population such as liposuction or free nipple procedures.
